# Mr. Scott's Case of Scrotal Hernia

**Published:** 1807-05

**Authors:** James Scott

**Affiliations:** Second Surgeon of his Majesyt's ship Rose. Off Brest


### *To the Editors of the Medical and Physical Journal,*

GENTLEMEN,

HAVING received much pleasure and instruction from your Medical and Physical Journal, and considering it the best channel through which informatioh can be conveyed to the medical world, I am induced to trouble you with a few observations on a case of Scrotal Hernia, that lately fell under my care. Should you consider them worth insertion in your valuable publication, I shall be encouraged in future communications of any thing remarkable, that may occur in my practice. For the present I deem it unnecessary to make any apology, as all sincere endeavours to establish medical facts and extend the science, must meet your approbation and patronage.

James Faruja, (a native of Malta, and seaman in this ship) a short stout man, ætat. 22, ruptured himself by a fall. On examination, the right side of the scrotum appeared enormously distended, and the left much enlarged. The contents of the tumor seem to have been omentum and intestine, as the swelling felt smooth in some places, but had many unequal surfaces; and during the operation of the taxis a considerable quantity of air was distinctly heard by the assistant and myself, passing to the belly, before the protruded parts could be returned into the abdomen.

What I conceive worthy of remark is, that as soon as the the Hernia, (which was on the right side) was completely reduced, the swelling of the left side of the scrotum entirely subsided: for, at first, I supposed the patient was ruptured on both sides, and consequently an unnecessary degree of alarm or rather pity was excited in my mind. On minutely examining the left side of the scrotum, I found that the testicle had been entirely absorbed (from what cause he knows not), and the spermatic vessels, and the coats of the testis only remained. This, I believe, uncommon swelling, shews to what an extent the septum scroti is capable of being stretched by pressure; and, I think, affords a good practical lesson in the palliative treatment of hydrocele tunicæ vaginalis testis, and in the radical cure by injection, when the disease exists in both testes, or is supposed to do so. For the case before us proves the possibility of the septum scroti yielding so as to admit extraneous bodies, though only on one side of it, to occupy almost the whole cavity of the scrotum, and, therefore, in vaginal hydrocele both testicles may appear to be affected, when only one is in reality diseased, by the fluid compressing the septum into the healthy cavity, so that an operator, unacquainted with this circumstance, might unhappily wound the septum, and occasion a very troublesome, if not fatal hæmorrhage from its vessels.

Again, if both testicles are affected, still this accident might happen, unless the fluids reciprocally balance each other, so as to retain the septum in its natural situation; and I am convinced great mischief has happened from unequal pressure, as we are so frequently warned by authors of the risque there is of wounding the septum with the trocar. Indeed, it is mentioned as a common cause of hæmatocele; and I suspect it is almost the only one. This collection of blood has also been supposed to arise from the vessels bursting, when the support of the fluid was removed, and that may sometimes be the case; but I am afraid a wound of the septum generally occasions it.

I would therefore humbly propose, that whenever the scrotum is to be punctured for hydrocele, and both sides are affected, or seem to be so, the trocar should be passed first through the largest side of the swelling, with the point inclined rather towards the thigh of that side; and, this being done, if the other cavity is found diseased also, the fluid may be drawn off with perfect safety, as the septum will then be forced towards the side whose contents have been evacuated.

This caution may, perhaps, at first sight appear unnecessary; but when it is considered that an operator unapprized of the danger, is just as likely to perforate the small swelling as the large one, first, I hope my observations will not be deemed impertinent.

I am, &c.

JAMES SCOTT,

Second Surgeon of his Majesyt's ship Rose.

*Off Brest, March* 4, 1807.

